# *Neorickettsia sennetsu* as a Neglected Cause of Fever in South-East Asia

**DOI:** 10.1371/journal.pntd.0003908

**Published:** 2015-07-09

**Authors:** Sabine Dittrich, Weerawat Phuklia, Gareth D. H. Turner, Sayaphet Rattanavong, Vilada Chansamouth, Stephen J. Dumler, David J. P. Ferguson, Daniel H. Paris, Paul N. Newton

**Affiliations:** 1 Lao-Oxford-Mahosot Hospital-Wellcome Trust Research Unit (LOMWRU), Microbiology Laboratory, Mahosot Hospital, Vientiane, Lao People's Democratic Republic; 2 Centre for Tropical Medicine and Global Health, Nuffield Department of Medicine, Churchill Hospital, University of Oxford, Oxford, England, United Kingdom; 3 Mahidol-Oxford Tropical Medicine Research Unit (MORU), Faculty of Tropical Medicine, Mahidol University, Bangkok, Thailand; 4 Departments of Pathology, Microbiology and Immunology University of Maryland School of Medicine, Baltimore, Maryland, United States of America; 5 Nuffield Department of Clinical Laboratory Science, John Radcliffe Hospital, University of Oxford, Oxford, United Kingdom; University of Texas Medical Branch, UNITED STATES

## Abstract

*Neorickettsia sennetsu* infection is rarely recognized, with less than 100 globally reported patients over the last 50 years. The disease is thought to be contracted by eating raw fish, a staple of many South-East Asian cuisines. In 2009, the first patient with sennetsu was identified in the Lao PDR (Laos), raising the question as to how common this organism and related species are in patients presenting with fever. We investigated the frequency of *N*. *sennetsu* infection at hospitals in diverse areas of Laos. Consenting febrile hospital inpatients from central (Vientiane: n = 1,013), northern (Luang Namtha: n = 453) and southern (Salavan: n = 171) Laos were screened by PCR for *N*. *sennetsu*, if no previous positive direct diagnostic test was available. A PCR-restriction fragment length polymorphism assay was developed to differentiate between *N*. *sennetsu*, *Ehrlichia chaffeensis* and *Anaplasma phagocytophilum*. To allow more detailed studies of *N. sennetsu*, culture was successfully established using a reference strain (ATCC VR-367), identifying a canine-macrophage cell line (DH82) to be most suitable to visually identify infection. After screening, *N*. *sennetsu* was identified and sequence confirmed in four (4/1,637; 0.2%) Lao patients. Despite the previously identified high seroprevalence of *N*. *sennetsu* antibodies in the Lao population (~17%), acute *N*. *sennetsu* infection with sufficient clinical signs to prompt hospitalization appears to be rare. The reservoir, zoonotic cycle and pathogenicity of *N*. *sennetsu* remain unclear and require further investigations.

## Author Summary

After the rediscovery of *Neorickettsia sennetsu* as a possible fish-borne cause of fever in 2009, it remained unclear to what extent this pathogen was responsible for disease in Laos and South-East Asia. The original reports from the 1960s proposed an epidemiological link between eating raw fish and sennetsu. Dishes prepared with raw or fermented fish are very commonly eaten in rural South-East Asia and the resulting burden of intestinal parasites is high. As laboratory and research capacity is limited in Laos we hypothesized that sennetsu may have been missed as a common cause of fever in the country. Therefore, our work focused on the establishment of a simple typing method for this and related organisms. We describe the screening of ~1,600 patients from urban and rural Laos, finding *N*. *sennetsu* in diverse regions of the country. We identified surprisingly small numbers by PCR in comparison to the previously described high seroprevalence. This underlines the importance for continued research into the diagnosis, pathophysiology and natural history of this unusual pathogen.

## Introduction

*Neorickettsia sennetsu* was not reported for 24 years prior to the identification of a Lao patient with sennetsu in 2009 [1]. This pathogen was the first documented cause of glandular fever (infectious mononucleosis) in Japan in 1954, characterized by fever, weakness, anorexia, lymphadenopathy and peripheral blood mononucleosis with atypical lymphocytes [1]. The route of human infection was thought to be the consumption of raw fish (gray mullet, *Mugil cephalus*) [1]. A recent study in rural, northern Thailand identified two additional acute infections of *N*. *sennetsu* in febrile patients by serology [2].

*N*. *sennetsu* is an intracellular bacterium which grows in host-membrane-lined cytoplasmic vacuoles and is a member of the family Anaplasmataceae and is closely related to *Ehrlichia* spp. and *Anaplasma* spp. [3]. All *Neorickettsia* spp. are thought to be transmitted by digeneans, parasitic flatworms which transmits the organisms vertically within the life cycle as well as horizontally to the animal or human host. In addition to sennetsu, pathogenic infections by *Neorickettsia* spp. include salmon poisoning disease (dog; *N*. *helminthoeca*) and Potomac horse fever (horse; *N*. *risticii*) [4,5]. A recent study suggests that *N*. *sennetsu* is phylogenetically more closely related to *N*. *risticii*, than *N*. *helminthoeca* [6]. Despite the molecular detection of only one patient with acute illness, serological data from Lao suggested that up to 17% of adults were exposed to *N*. *sennetsu* [1]. The ingestion of raw, undercooked and fermented fish is an important part of Lao culture associated with social events and religious celebrations, with little knowledge about the risks associated with raw foods [7]. The resulting public health problem is illustrated by the high prevalence of intestinal parasites transmitted via undercooked fish [7–9]. The presence of both high parasitic disease burdens and high sennetsu seroprevalence in the population suggests that *N*. *sennetsu* could be an under recognized cause of fever [10]. The detection of *N*. *sennetsu* in a febrile Lao patient at our hospital, as well as in a single fish (*Anabas testinudeus*), highlighted unanswered questions regarding the prevalence, geographical distribution, zoonotic cycle, risk factors and pathogenicity of this rare pathogen [10]. As laboratory capacity in Laos and adjacent countries is scarce, sennetsu patients are unlikely to be diagnosed. The current study aimed to determine the hospital incidence of sennetsu in a large series of febrile patients from different geographical locations in Laos. Furthermore, we describe the development of tools to allow simple post-PCR identification as well as successful propagation of *N*. *sennetsu* in an endemic area, aiding timely public health interventions and future research on this organism [10,11].

## Materials and Methods

**Fig 1:**
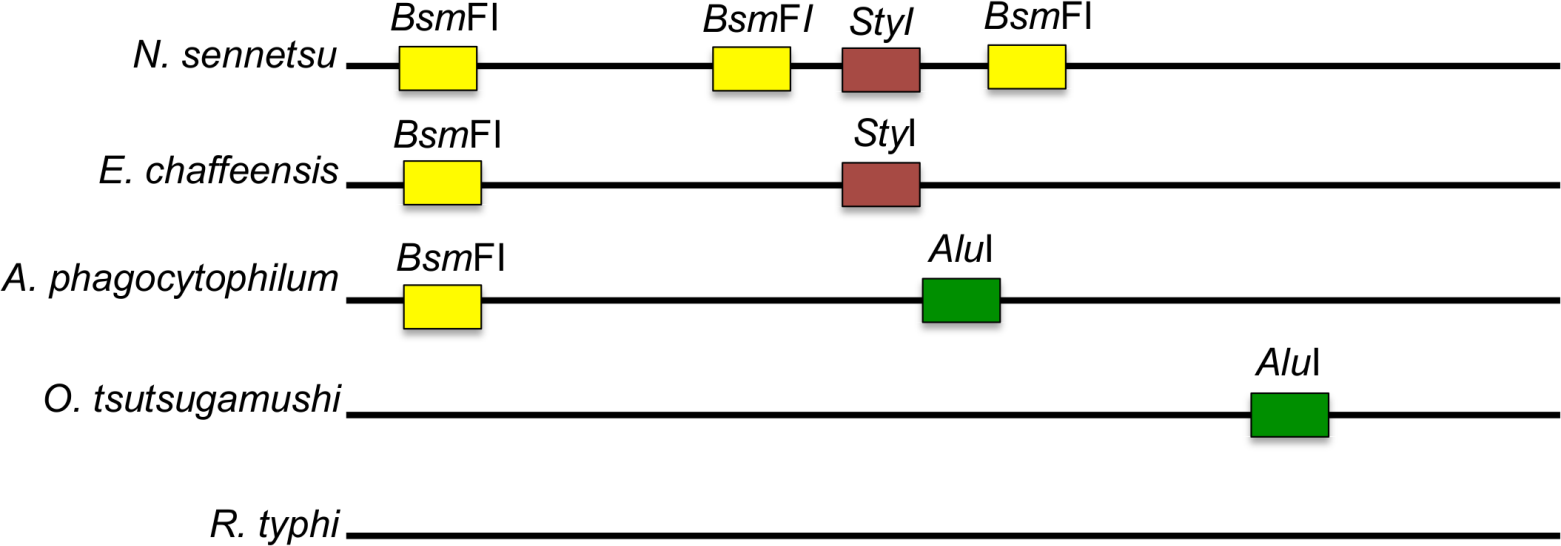
Schematic alignment of *N*. *sennetsu* and related organisms showing the restrictions sites used for the RFLP. The unique RFLP pattern of the 16 sRNA gene target, after incubation with *Alu*I (green), *Bsm*FI (yellow) or *Sty*I (red) allow the differentiation of *N*. *sennetsu*, *E*. *chaffeensis* and *A*. *phagocytophium* as well as other potentially amplified organisms. The resulting fragment sizes are as follows; *N*. *sennetsu*–*Alu*I: 345bp (uncut); *Bsm*F1: 180bp, 80bp, 60bp, 16bp; *Sty*I: 215bp, 127bp; *E*. *chaffeensis–Alu*I: 345bp (uncut); *Bsm*F1: 328bp, 16bp; *Sty*I: 215bp, 127bp; *A*. *phagocytophium–Alu*I: 199bp, 145bp *Bsm*F1: 328bp, 16bp; *Sty*I: 345bp (uncut).

**Fig 2:**
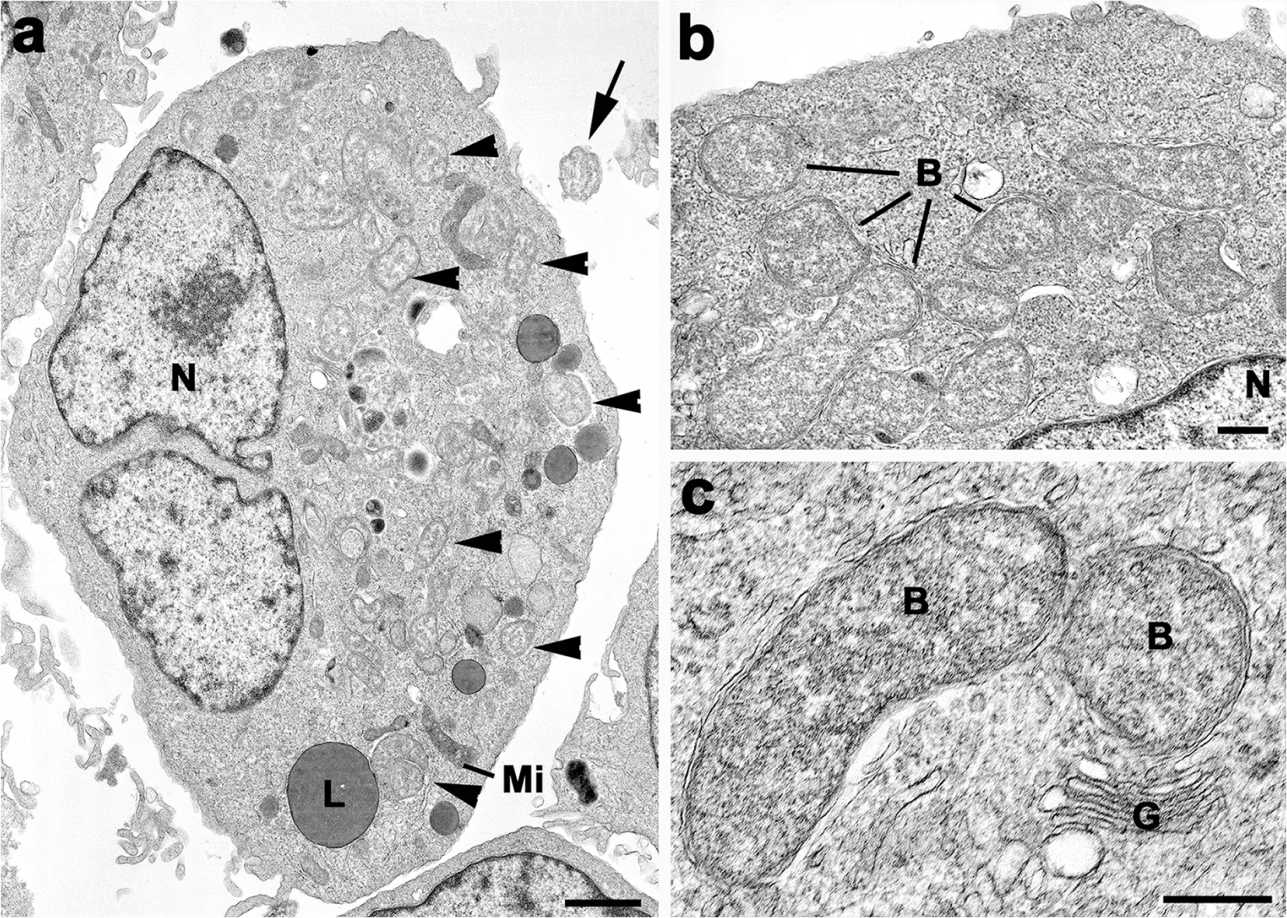
Electron microscopic appearance of *N*. *sennetsu* (ATCC VR-367, Miyayama strain) in DH82 canine monocyte cultures. a) Low power micrograph of an infected cell in which a number of bacteria can be identified in the cytoplasm (arrowheads) in addition to the nucleus (N), mitochondria (Mi) and lipid droplet (L). Note the single extracellular bacterium (arrow). Bar represents 1μm. **b)** Enlargement of part of the cytoplasm showing a number of gram negative bacteria (B). N–nucleus. Bar represents 200nm. **c)** Detail of N. sennetsu (arrow) showing the gram negative bacteria limited by two unit membranes located within a membrane bound vacuole. G—Golgi stack. Bar represents 200nm.

Prospectively collected samples from consecutive patients were submitted to the Mahosot Hospital Microbiology Laboratory (n = 1,013) from Vientiane (VTE; longitude 102.6119°E, latitude 17.96‟04°N) admitted between January 2010 to December 2011 with suspected typhus to Mahosot Hospital (n = 816), Friendship Hospital (n = 29), Settathirat Hospital (n = 167) and the Military Hospital (n = 1). All patients presented with fever and local physicians suspected typhus. Mahosot Hospital and the four other major hospitals in VTE (1,210 beds total) serve a population of ~900,000 people, including the urban population of Vientiane City and surrounding farming communities of Vientiane Province. Patients from the southern and northern provinces (Salavan Provincial Hospital [SV], longitude 106.2500°E, latitude 15.4300° N; Luang Namtha Provincial Hospital [LNT], longitude 101.4025°E, latitude 20.9606° N) were included from May 2008–December 2010 as part of a large undifferentiated fever study [12]. They were included in the current investigation if they had previously not been assigned a direct (culture/PCR) diagnosis [12]. All samples were screened using a described conventional PCR targeting the 16S rRNA gene, which amplifies *N*. *sennetsu*, *A*. *phagocytophilum*, *E*. *chaffeensis* and related organisms, with some amendments [1,13]. PCR reactions were set up in a total of 25 μL with 1x Platinum UDG-Supermix (Invitrogen) with annealing temperature of 61°C. Positive control-DNA from *N*. *sennetsu*, *A*. *phagocytophilum* and *E*. *chaffeensis* cultures as well as no-template controls, were added in each run. DNA was extracted from 200 μL EDTA buffy coat as described [12]. All positive samples were confirmed using PCRs targeting *glt*A and *omp*85, as described [1]. Samples positive for all three targets were processed for DNA sequencing (Macrogen, South Korea) and subsequently analyzed using NCBI-BLAST. To differentiate *N*. *sennetsu*, *A*. *phagocytophilum* and *E*. *chaffeensis* without the need for third party-sequencing, a simple restriction fragment length polymorphism (RFLP) assay on the 16 sRNA amplicon [13] was developed (Fig 1). Ten microliters of PCR product containing dTTP, was incubated with *Alu*I (NEB, USA; 1 Unit, 37°C, 2h), *Sty*I (NEB, USA; 1 Unit, 37°C, 2h), and *Bsm*F1 (NEB, USA; 1 Unit, 65°C, 2h); and the appropriate reaction buffer. PCR amplicons and RFLP products were visualized on a 4% agarose gel (NuSieve GTG Agarose, Lonza) and RFLP-patterns compared to positive controls (S2 Fig and S1 Table). To further allow characterization and genotyping of patients, *N*. *sennetsu* (strain Miyayama) culture was established using the ATCC VR-367 reference strain. It was propagated at biosafety level-3 in an adherent canine macrophage-like cell line (DH82; ATCC CRL-10389), African green monkey kidney cells (VERO; provided by the Australian Rickettsia Reference Laboratory, Geelong, Australia) and mouse fibroblast cell line (L929; provided by the Australian Rickettsia Reference Laboratory, Geelong, Australia). Eagle’s Minimum Essential Medium (EMEM, Invitrogen, UK) was supplemented with 10% fetal bovine serum (FBS; Invitrogen, UK) and 2 mM L-glutamine (Invitrogen, UK) for DH82 cells. RPMI-1640 (Pacific Science, Thailand) was supplemented with 10% FBS (Invitrogen, UK) for VERO and L929 cells and cultures were maintained as described [14,15]. Cultures were observed daily for cytopathic effect (CPE) and a weekly PCR [13] on supernatant and cells was performed. Confirmation of intracellular growth was obtained by examining cell samples by electron microscopy (EM; Fig 2). Confluent layers of infected cells were trypsinised, washed with 1xPBS and than fixed with 2.5% glutaraldehyde, in 0.1M phosphate buffer. The samples were post-fixed in 2% osmium tetroxide in phosphate buffer and dehydration in ethanol followed by treatment with propylene oxide, prior to embedding in TABB epoxy resin. Thin sections of suitable areas were examined using a Jeol 1200EX electron microscope [16].

Using the optimized DH82 culture protocol, growth from buffy coat (n = 2) and serum samples (n = 2; 200 μL) from two patients with PCR confirmed *N*. *sennetsu* infection was attempted. Samples had been frozen at -80°C (without cryopreservative) for up to two years prior to inoculation. Cultures were observed daily for CPE and tested weekly by PCR (supernatant/cells) for 90 days. DNA sequence analysis was performed using CLC Main Workbench 7.0.

### Ethics statement

All study patients provided written informed consent prior to sample collection, if minors were participants, a parent or guardian of any child participant provided informed consent on their behalf. Ethical approval for all investigations was granted by OXTREC (015–10 and 006–07 University of Oxford, UK) and the Faculty of Medical Sciences Committee (University of Health Sciences, Lao PDR).

## Results

In total, 1,637 febrile patient samples were included from three different geographical locations in Laos, LNT in the north (n = 453), SV in the south (n = 171) and VTE in the center (n = 1,013). The majority of patients were male (56.2%) with a median age of 25 years (range: 1–96). The predominant symptoms of patients at all sites were fever, headache and myalgia. Across the three locations 10.6%-50.7% of patients had lymphadenopathy and 12.2%-28.7% of patients had pharyngitis. Peripheral blood atypical lymphocytes were not determined. All available patient samples were screened and strong positive bands at the correct size were observed in four of 1,642 samples (0.24%) and in addition 75 samples showed weak positive bands (75/1642; 4.6%). Positive and negative controls gave the correct results in all experiments. The strong positive results could be confirmed as *N*. *sennetsu* by *glt*A, *omp*85 PCR, 16s RNA sequencing as well as the 16 sRNA-RFLP. DNA sequences were deposited on Genbank (Accession Numbers: KM355198-KM355200; LNT554_fragment) (S2 Table and S1 Fig). The remaining weak positive samples were considered negative, as all subsequent confirmatory tests (*glt*A and *omp*B-PCR, PCR-16 sRNA RFLP [selected samples only] or sequencing) were negative. The newly established PCR-RFLP assay showed 100% concordance with the sequencing result for *N*. *sennetsu* in strongly positive samples (S2 Fig). Due to a lack of *A*. *phagocytophilum* and *E*. *chaffeensis* positive patient samples, the ability of the PCR-RFLP assay to correctly identify those pathogens in patients rather than positive controls could not be determined.

Of the four *N*. *sennetsu* patients, two (2/1,013; 0.2%) were from central Laos, one from the north (1/453; 0.2%) and one from the south (1/171; 0.6%). All presented with fever and headache without focal signs of infection (Table 1). Both patients from VTE were hospitalized while those from the south and north were treated as outpatients (Table 1). No seasonal clustering was observed. For three patients no other cause of infection was identified, but patient_4 (SV) was also IgM-positive for dengue in the convalescent sample (ELISA), suggestive of an acute infection [12]. Treatment was unknown for two of the patients, one was treated with co-trimoxazole and ampicillin, both not expected to be effective against *N*. *sennetsu*, and one with ofloxacin, which is expected to be active against *N*. *sennetsu* [1,17]. Three patients were well 12, 14 and 75-months post infection whilst the fourth patient (SV) was lost to follow up.

**Table 1:** Overview of confirmed *N*. *sennetsu* patients in Laos.

	Patient 1 (Vientiane)	Patient 2 (Vientiane)	Patient 3 (Luang Namtha)	Patient 4 (Salavan)
**General information**	Female, 42 years Infectious Disease Ward (Mahosot Hospital) January 2010 No antibiotics prior to admission	Male, 22 years Internal Medicine Ward (Settathirat Hospital) May 2010 No antibiotics prior to admission	Male, 28 years Outpatient September 2008 Amoxicillin prior to visit	Male, 23 years Outpatient February 2009 Ampicillin prior to visit
**Symptoms**	9 days ill, Fever, Headache, Arthralgia, Myalgia	7 days ill, Fever, Headache, Arthralgia, Myalgia	2 days ill, Headache, Cough	4 days ill, Headache, Chill, Arthralgia, Myalgia, Backpain, Retro-orbital pain
**Reported environmental exposure (2 weeks prior)**	Rice field/Forest/ Fishing rats/tick/flea/cat	Rats	n.a.	Rice field
**Recent raw fish consumption**	Yes	Yes	Yes	Yes
**Physical examination**	Temperature 37.5°C Pulse: 110 /min, Blood pressure: 100/60 mmHg, Respiratory rate: 24/min, Glasgow Coma Sclae (GCS): 15/15	Temperature 38.5°C, Pulse: 90 /min, Blood pressure: 170/70 mmHg, Respiratory rate: 20/min, GCS: 15/15, Enlarged cervical and right inguinal lymph nodes	Temperature 39.0°C, Rash, Pharyngeal Erythema, GCS: 15/15	Temperature 38.5°C, Pulse: 100 /min, Blood pressure: 120/80 mmHg, Respiratory rate: 20/min, GCS: 15/15
**Laboratory findings (blood)**	Haematocrit 43%, White blood cells 11.8x10^9^/L, Platelets 310x10^9^/L	Haematocrit 36.9%, Haemgologin 129 g/L, White blood cells 4.81x10^9^/L, Polymorphs 51.5%, Lymphocytes 36.6%, Platelets 182x10^9^/L, Glucose 5.4 mmoL/L, Creatinine 88.4 μmol/L, AST 127 IU/L, ALT 120 IU/L	White blood cells 5.7x10^9^ /L, Lymphocytes 57%, Monocytes 3%, Eosinophils 1%, C-reactive protein (CRP) 87 mg/L	CRP 10.3 mg/L
**Diagnostic findings**	Malaria smear: negative, Haemoculture [20]: no growth *O*. *tsutsugamushi/Rickettsia* spp.*/Leptospira* spp. qPCR [12]: negative	Abdominal ultrasound: normal, Malaria smear: negative, Haemoculture [20]: no growth *O*. *tsutsugamushi/Rickettsia* spp.*/Leptospira* spp. qPCR [12]: negative	Malaria smear/PCR [12]: negative, Haemoculture [20]: no growth O. *tsutsugamushi*/*Rickettsia* spp./*Leptospira* spp. qPCR [12]: negative, Dengue qPCR [12]: negative, Dengue ELISA (IgM) [12]: negative, Scrub/murine typhus IFA [12]: negative, Leptospira MAT [12]: negative	Malaria smear&PCR [12]: negative, Haemoculture [20]: no growth *O*. *tsutsugamushi*/*Rickettsia* spp./*Leptospira* spp. qPCR [12]: negative, Dengue qPCR [12]: negative, Dengue ELISA (IgM) [12]: positive Dengue ELISA (IgM) [12]: positive, Dengue ELISA (NS1) [12]: negative, Scrub/murine typhus IFA [12]: negative, Leptospira MAT [12]: negative
**Treatment/Outcome**	Not available, Discharged well	Rehydration, Paracetamol, Ofloxacin, Discharged well	Amoxicillin, Co-trimoxazole, Discharged well	Not available, Discharged well

*N*. *sennetsu* reference strain (ATCC VR-367) was successfully established in Vero, L929 and DH82 cells and intracellular growth confirmed by EM and bacteria identified in the cytoplasm of the host cell (Fig 2A). At higher magnification the bacteria could be seen to be located in membrane bound vacuoles and were limited by the double unit membrane characteristic for gram-negative bacteria (Fig 2B and 2C). In contrast to Vero and L929, the canine cell line DH82 showed distinct CPE on day 3 post-infection with the reference strain, a phenomenon not previously described. Daily visual observation of the cultures inoculated with patient specimens did not show a CPE up to 90 days. Further, weekly cell and supernatant samples were negative for *N*. *sennetsu* by PCR up to 90 days.

## Discussion

Large-scale retrospective screening of febrile patients, resulted in the identification of four patients with sennetsu, from three disparate Lao geographical areas, raising the total number of patients recorded in the country to five. All patients had a history of eating raw fish consistent with the suggested route of transmission. Still as eating raw fish is very common in Laos [1] this does not represent a strong epidemiological link.

Despite the optimization of culture conditions in this first comparative study of different cell lines, propagation of the pathogen from patient samples was unsuccessful and precluded further pathogenicity and genomics studies. The overall very low frequency of detected *N*. *sennetsu* was unexpected considering an estimated seroprevalence of ~17% in Lao patients and the significant burden of other fish-borne diseases in the country [1,7–9]. This underlines the need for further investigations to elucidate if raw fish and their trematodes are indeed the mode of transmission for this pathogen. The discrepancy between high serological prevalence and the relative rarity of disease could also be explained by a very long persisting antibody responses, high frequency of asymptomatic infections or symptoms not prompting hospital admission, short duration of illness, low pathogen blood density, serological cross-reaction with other *Neorickettsia* species and other Anaplasmataceae, or that the serological cut-off used for Laos previously (1:100) was too low. The first patients were identified in Japan, on Kyushu in the 1950s on the basis of clinical observations and propagation of *N*. *sennetsu* in animals and not by antibody-based or PCR-based tests [18].

Important limitations of this study include that samples came from patients with fever rather than those with infectious mononucleosis and data regarding atypical lymphocytosis was not obtained. The observed weak positive samples were most likely false positives due to weak priming of bacterial but non-sennetsu DNA. However, it cannot be ruled out entirely that these samples contained *N*. *sennetsu* in quantities too low to be confirmed with our single PCR assays. PCR carryover, or genomic DNA contamination as a source of low positivity is made unlikely by strict physical separation of working areas (pre-PCR, sample preparation, post-PCR, electrophoresis rooms) as well as the use of Uracil DNA glycosylase/dUTPs in all screening PCRs [19]. Unsuccessful propagation was possibly due to extended storage of samples prior to inoculation and the resulting loss of viability common in Anaplasmataceae (S. Dumler, personal observation).

In conclusion, amongst patients in diverse environments in Laos *N*. *sennetsu* was not identified as a major cause of fever using molecular detection methods. However, it will be important to continue similar investigations in patients with suspected infectious mononucleosis. This study further highlights significant knowledge gaps regarding the pathogen reservoir, mode of transmission, disease kinetic and optimal diagnostic techniques of this unusual organism. The role of *N*. *sennetsu* as a pathogen and the biology of this rare disease requires further multidisciplinary investigations.

## Supporting Information

S1 Checklist STROBE checklist. (DOCX)

S1 Table Fragment sizes after RLFP with *Alu*I, *Bsm*FI and *Sty*I, to differentiate between *N*. *sennetsu*, *Ehrlichia* spp. or *Anaplasma* spp. (DOCX)

S2 Table LNT554 sequence fragment after removal of primer site. (DOCX)

S1 Fig Sequence alignment of all patient (GenBank Accession Numbers: KM355198-KM355200) samples with the *N*. *sennetsu* reference strain. Alignment includes primers, to illustrate that primer sequences were removed from the submitted and analysed sequence. Matched residues are shown with a dot (.) and missing sequences are indicated with a hyphen (-).
(DOCX)

S2 Fig Visualized fragments after RFLP to differentiate between *N*. *sennetsu*, *Ehrlichia spp* or *Anaplasma spp*. A) Depicting RFLP result using positive controls. Lane 1: Size ladder (Hyperladder II, Bioline); Lane 2: *N*. *sennetsu* result after digest with *Alu*I; Lane 3: *N*. *sennetsu* result after digest with *Bsm*F I*; Lane 4: *N*. *sennetsu* result after digest with *Sty*I*; Lane 5: *Ehrlichia* spp. result after digest with *Alu*I; Lane 6: *Ehrlichia* spp. result after digest with *Bsm*FI; Lane 7: *Ehrlichia* spp. result after digest with *Sty*I; Lane 8: *Anaplasma* spp. result after digest with *Alu*I*; Lane 9: *Anaplasma* spp. result after digest with *Bsm*F I; Lane 10: *Anaplasma* spp. result after digest with *Sty*I; B) Depicting RFLP results of patient 1–4. Lane 1: Size ladder (Hyperladder II, Bioline); Lane 2: Patient 1 result after digest with *Alu*I; Lane 3: Patient 1 result after digest with *Bsm*F I*; Lane 4: Patient 1 result after digest with *Sty*I*; Lane 5: Patient 2 result after digest with *Alu*I; Lane 6: Patient 2 result after digest with *Bsm*FI*; Lane 7: Patient 2 result after digest with *Sty*I*; Lane 8: Patient 3 result after digest with *Alu*I; Lane 9: Patient 3 result after digest with *Bsm*F I*; Lane 10: Patient 3 result after digest with *Sty*I*; Lane 11: Patient 4 result after digest with *Alu*I; Lane 12: Patient 4 result after digest with *Bsm*F I*; Lane 13: Patient 4 result after digest with *Sty*I*;
(DOCX)
