# Supplementary material for: Neorickettsia sennetsu as a Neglected Cause of Fever in South-East Asia
Source: PLoS Negl Trop Dis. 2015 Jul 9;9(7):e0003908. doi: 10.1371/journal.pntd.0003908 (PMC4497638; doi:10.1371/journal.pntd.0003908)
Supplement: S1 Checklist — (DOCX) [file pntd.0003908.s001.docx]

|  | Item No | Recommendation |
| --- | --- | --- |
| **Title and abstract** | x | (*a*) Indicate the study’s design with a commonly used term in the title or the abstract |
|  |  | (*b*) Provide in the abstract an informative and balanced summary of what was done and what was found |
| Introduction | | |
| Background/rationale | x | Explain the scientific background and rationale for the investigation being reported |
| Objectives | x | State specific objectives, including any prespecified hypotheses |
| Methods | | |
| Study design | x | Present key elements of study design early in the paper |
| Setting | x | Describe the setting, locations, and relevant dates, including periods of recruitment, exposure, follow-up, and data collection |
| Participants | x | (*a*) *Cohort study*—Give the eligibility criteria, and the sources and methods of selection of participants. Describe methods of follow-up  *Case-control study*—Give the eligibility criteria, and the sources and methods of case ascertainment and control selection. Give the rationale for the choice of cases and controls  *Cross-sectional study*—Give the eligibility criteria, and the sources and methods of selection of participants |
|  |  | (*b*) *Cohort study*—For matched studies, give matching criteria and number of exposed and unexposed  *Case-control study*—For matched studies, give matching criteria and the number of controls per case |
| Variables | n.a. | Clearly define all outcomes, exposures, predictors, potential confounders, and effect modifiers. Give diagnostic criteria, if applicable |
| Data sources/ measurement | n.a. | For each variable of interest, give sources of data and details of methods of assessment (measurement). Describe comparability of assessment methods if there is more than one group |
| Bias | x | Describe any efforts to address potential sources of bias |
| Study size | x | Explain how the study size was arrived at |
| Quantitative variables | n.a. | Explain how quantitative variables were handled in the analyses. If applicable, describe which groupings were chosen and why |
| Statistical methods | n.a. | (*a*) Describe all statistical methods, including those used to control for confounding |
|  |  | (*b*) Describe any methods used to examine subgroups and interactions |
|  |  | (*c*) Explain how missing data were addressed |
|  |  | (*d*) *Cohort study*—If applicable, explain how loss to follow-up was addressed  *Case-control study*—If applicable, explain how matching of cases and controls was addressed  *Cross-sectional study*—If applicable, describe analytical methods taking account of sampling strategy |
|  |  | (*e*) Describe any sensitivity analyses |
