# Supplementary material for: Neorickettsia sennetsu as a Neglected Cause of Fever in South-East Asia
Source: PLoS Negl Trop Dis. 2015 Jul 9;9(7):e0003908. doi: 10.1371/journal.pntd.0003908 (PMC4497638; doi:10.1371/journal.pntd.0003908)
Supplement: S1 Table — (DOCX) [file pntd.0003908.s002.docx]

**Table S1: Fragment sizes after RLFP with *Alu*I, *Bsm*FI and *Sty*I, to differentiate between *N. sennetsu*, *Ehrlichia* spp. or *Anaplasma* spp.**

|  |  | *N. sennetsu* | *Ehrlichia* spp. | *Anaplasma* spp. |
| --- | --- | --- | --- | --- |
| Restriction Enzyme | *Alu*I | 344 bp | 344 bp | 199 bp  145 bp |
|  | *Bsm*FI | 180 bp  80 bp  60 bp  16 bp | 328 bp  16 bp | 328 bp  16 bp |
|  | *Sty*I | 215 bp  127 bp | 216 bp  127 bp | 344bp |
