# Supplementary material for: Neorickettsia sennetsu as a Neglected Cause of Fever in South-East Asia
Source: PLoS Negl Trop Dis. 2015 Jul 9;9(7):e0003908. doi: 10.1371/journal.pntd.0003908 (PMC4497638; doi:10.1371/journal.pntd.0003908)
Supplement: S2 Table — (DOCX) [file pntd.0003908.s003.docx]

**Table S2: LNT554 sequence fragment after removal of primer site.**

| Sequence name | Sequence | Target gene | Length |
| --- | --- | --- | --- |
| LNT554_fragment | CGGCTAACTCCGTGCCAGCAGGCGCCGTCATAAGAAGGGGGGTGGCCTT | 16sRNA | 49bp |
