# Supplementary figures and images for: Neorickettsia sennetsu as a Neglected Cause of Fever in South-East Asia
Source: PLoS Negl Trop Dis. 2015 Jul 9;9(7):e0003908. doi: 10.1371/journal.pntd.0003908 (PMC4497638; doi:10.1371/journal.pntd.0003908)

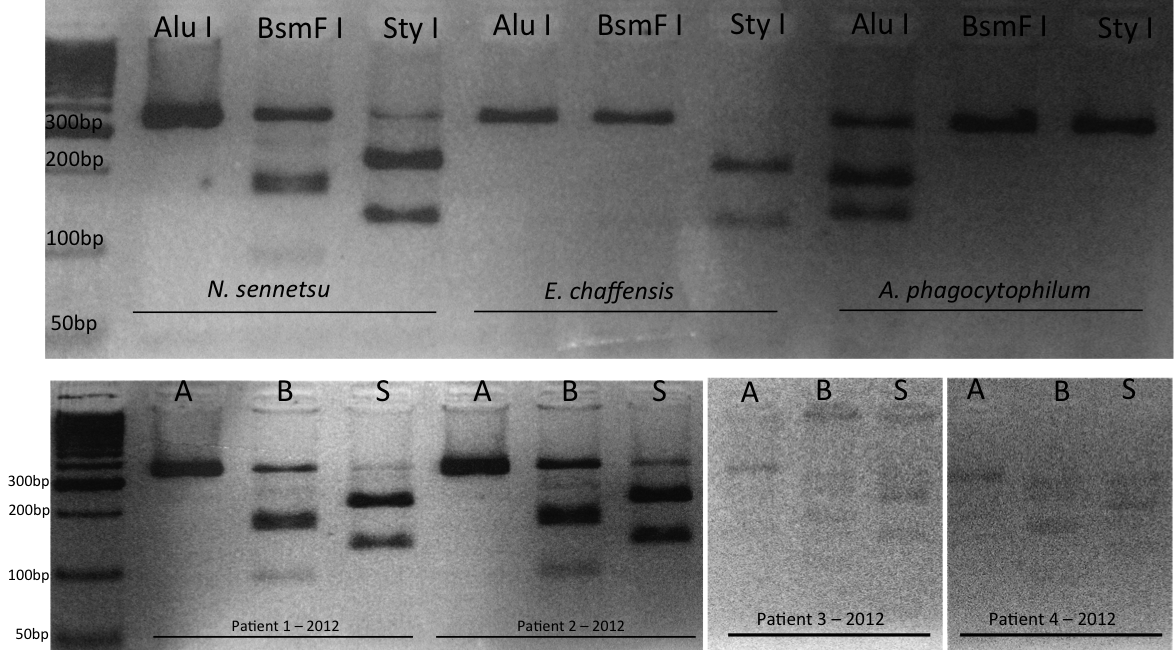

Supplement: S2 Fig — A) Depicting RFLP result using positive controls. Lane 1: Size ladder (Hyperladder II, Bioline); Lane 2: N. sennetsu result after digest with AluI; Lane 3: N. sennetsu result after digest with BsmF I*; Lane 4: N. sennetsu result after digest with StyI*; Lane 5: Ehrlichia spp. result after digest with AluI; Lane 6: Ehrlichia spp. result after digest with BsmFI; Lane 7: Ehrlichia spp. result after digest with StyI; Lane 8: Anaplasma spp. result after digest with AluI*; Lane 9: Anaplasma spp. result after digest with BsmF I; Lane 10: Anaplasma spp. result after digest with StyI; B) Depicting RFLP results of patient 1–4. Lane 1: Size ladder (Hyperladder II, Bioline); Lane 2: Patient 1 result after digest with AluI; Lane 3: Patient 1 result after digest with BsmF I*; Lane 4: Patient 1 result after digest with StyI*; Lane 5: Patient 2 result after digest with AluI; Lane 6: Patient 2 result after digest with BsmFI*; Lane 7: Patient 2 result after digest with StyI*; Lane 8: Patient 3 result after digest with AluI; Lane 9: Patient 3 result after digest with BsmF I*; Lane 10: Patient 3 result after digest with StyI*; Lane 11: Patient 4 result after digest with AluI; Lane 12: Patient 4 result after digest with BsmF I*; Lane 13: Patient 4 result after digest with StyI*; (DOCX) [file pntd.0003908.s005.docx]
