# Supplementary material for: Neorickettsia sennetsu as a Neglected Cause of Fever in South-East Asia
Source: PLoS Negl Trop Dis. 2015 Jul 9;9(7):e0003908. doi: 10.1371/journal.pntd.0003908 (PMC4497638; doi:10.1371/journal.pntd.0003908)
Supplement: Supplementary file 5 [file pntd.0003908.s004.docx]

```
                                                       20          40          60
                                                        |           |           |
Neorickettsia sennetsu (gi|343206156) TACCCACAGA AGAAGTCCCG GCTAACTCCG TGCCAGCAGC CGCGGTAATA CGGAGGGGGC 60
                 Fwd_primer(partial) ....Y..... ........-- ---------- ---------- ---------- ---------- 18
                     LNT544_fragment ---------- --------.. .......... .......... ...C..C... A.A....... 42
                              ST1659 ---------- ---------. .......... .......... .......... .......... 41
                             UI15912 ---------- ---------- ---------- ---------- ---------- ---------- –
                               SV138 ---------- ---------- ---------- ---------- ---------- ---------- –
                          Rev_primer ---------- ---------- ---------- ---------- ---------- ---------- –

                                                       80         100         120
                                                        |           |           |
Neorickettsia sennetsu (gi|343206156) TAGCGTTGTT CGGAATTACT GGGCGTAAAG GGTGCGTAGG CGGTCCCGTA AGTTAGGTGT 120
                 Fwd_primer(partial) ---------- ---------- ---------- ---------- ---------- ---------- 18
                     LNT544_fragment .G.....--- ---------- ---------- ---------- ---------- ---------- 49
                              ST1659 .......... .......... .......... .......... .......... .......... 101
                             UI15912 ---------- ---------- --........ .......... .......... .......... 38
                               SV138 ---------- ---------- ---------- ---------- ---------- ---------- –
                          Rev_primer ---------- ---------- ---------- ---------- ---------- ---------- –

                                                      140         160         180
                                                        |           |           |
Neorickettsia sennetsu (gi|343206156) GAAATCCTTG GGCTTAACCC AGGAACTGCA TTTAAAACTG CGGGACTCGA ACGTGAGAGA 180
                 Fwd_primer(partial) ---------- ---------- ---------- ---------- ---------- ---------- 18
                     LNT544_fragment ---------- ---------- ---------- ---------- ---------- ---------- 49
                              ST1659 .......... .......... .......... .......... .......... .......... 161
                             UI15912 .......... .......... .......... .......... .......... .......... 98
                               SV138 -......... .......... .......... .......... .......... .......... 59
                          Rev_primer ---------- ---------- ---------- ---------- ---------- ---------- –

                                                      200         220         240
                                                        |           |           |
Neorickettsia sennetsu (gi|343206156) GGGCAATGGA ATTTTTGGTG TAGGGGTGAA ATCCGTAGAT ATCAAGAGGA ACGTCAGGGG 240
                 Fwd_primer(partial) ---------- ---------- ---------- ---------- ---------- ---------- 18
                     LNT544_fragment ---------- ---------- ---------- ---------- ---------- ---------- 49
                              ST1659 .......... .......... .......... .......... .......... .......... 221
                             UI15912 .......... .......... .......... .......... .......... .......... 158
                               SV138 .......... .......... .......... .......... .......... .......... 119
                          Rev_primer ---------- ---------- ---------- ---------- ---------- ---------- –

                                                      260         280         300
                                                        |           |           |
Neorickettsia sennetsu (gi|343206156) CGAAAGCGAT TGCCTGGATC ACAGTTGACG CTGAGGCACG AAAGCGTGGG GAGCAAACAG 300
                 Fwd_primer(partial) ---------- ---------- ---------- ---------- ---------- ---------- 18
                     LNT544_fragment ---------- ---------- ---------- ---------- ---------- ---------- 49
                              ST1659 .......... .......... .......... .......... .......... -........- 279
                             UI15912 .......... .......... .......... .......... .......... -......... 217
                               SV138 .......... .......... .......... .......... .......... .......... 179
                          Rev_primer ---------- ---------- ---------- ---------- ---------- ---------- –

                                                      320         340
                                                        |           |
Neorickettsia sennetsu (gi|343206156) GATTAGATAC CCTGGTAGTC CACGCTGTAA ACGATGAGTG TTAA 344
                 Fwd_primer(partial) ---------- ---------- ---------- ---------- ---- 18
                     LNT544_fragment ---------- ---------- ---------- ---------- ---- 49
                              ST1659 ---------- ---------- ---------- ---------- ---- 279
                             UI15912 ..-------- ---------- ---------- ---------- ---- 219
                               SV138 .....A.... .......... ...------- ---------- ---- 202
                          Rev_primer ---------- ---------- ---....... .......... C... 21
```
